# A Simple and Expeditious Route to Phosphate-Functionalized, Water-Processable Graphene for Capacitive Energy Storage

**DOI:** 10.1021/acsami.1c12135

**Published:** 2021-11-09

**Authors:** Edgar H. Ramírez-Soria, Sergio García-Dalí, Jose M. Munuera, Daniel F. Carrasco, Silvia Villar-Rodil, Juan M. D. Tascón, Juan I. Paredes, José Bonilla-Cruz

**Affiliations:** † Advanced Functional Materials & Nanotechnology Group, Centro de Investigación en Materiales Avanzados S. C. (CIMAV-Unidad Monterrey), Av. Alianza Norte 202, Autopista Monterrey-Aeropuerto Km 10, PIIT, Apodaca, Nuevo León C.P. 66628, México; ‡ Instituto de Ciencia y Tecnología del Carbono, INCAR-CSIC, C/Francisco Pintado Fe 26, Oviedo 33011, Spain

**Keywords:** phosphate-functionalized graphene, anodic exfoliation, capacitive energy storage

## Abstract

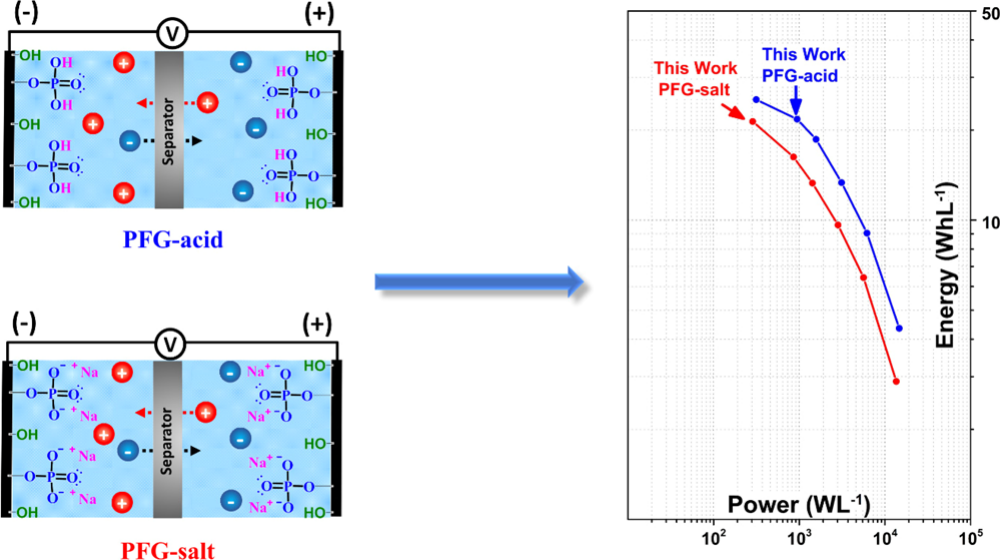

Phosphate-functionalized carbon-based nanomaterials have attracted significant attention in recent years owing to their outstanding behavior in electrochemical energy-storage devices. In this work, we report a simple approach to obtain phosphate-functionalized graphene (PFG) via anodic exfoliation of graphite at room temperature with a high yield. The graphene nanosheets were obtained via anodic exfoliation of graphite foil using aqueous solutions of H_3_PO_4_ or Na_3_PO_4_ in the dual role of phosphate sources and electrolytes, and the underlying exfoliation/functionalization mechanisms are proposed. The effect of electrolyte concentration was studied, as low concentrations do not lead to a favorable graphite exfoliation and high concentrations produce fast graphite expansion but poor layer-by-layer delamination. The optimal concentrations are 0.25 M H_3_PO_4_ and 0.05 M Na_3_PO_4_, which also exhibited the highest phosphorus contents of 2.2 and 1.4 at. %, respectively. Furthermore, when PFG-acid at 0.25 M and PFG-salt at 0.05 M were tested as an electrode material for capacitive energy storage in a three-electrode cell, they achieved a competitive performance of ∼375 F/g (540 F/cm^3^) and 356 F/g (500 F/cm^3^), respectively. Finally, devices made up of symmetric electrode cells obtained using PFG-acid at 0.25 M possess energy and power densities up to 17.6 Wh·kg^–1^ (25.3 Wh·L^–1^) and 10,200 W/kg; meanwhile, PFG-salt at 0.05 M achieved values of 14.9 Wh·kg^–1^ (21.3 Wh·L^–1^) and 9400 W/kg, with 98 and 99% of capacitance retention after 10,000 cycles, respectively. The methodology proposed here also promotes a circular-synthesis process to successfully achieve a more sustainable and greener energy-storage device.

## Introduction

Sustainable energy storage has recently arisen as a need to satisfy the global energy demand in the near future.^1^ The energy produced from any kind of energy source (solar, wind, hydro, tidal, geothermal, biomass) will require suitable and high-performance energy-storage devices.^2−5^ In this sense, several research studies have focused on studying the potential applications that graphene nanosheets have in electrochemical energy-storage devices, such as supercapacitors, metal ion batteries, metal-air batteries, etc.^6^ As is well known, a graphene layer is one of the thinnest known materials,^7^ comprising of a single layer of sp^2^ carbon atoms packed in a two-dimensional honeycomb lattice.^8^ Its remarkable mechanical,^9^ electronic,^10^ optical, and thermal^11^ properties have attracted a huge worldwide interest both in the scientific community and the industry. Nonetheless, producing graphene nanosheets at a mass scale and low cost is still a big challenge. Many works have studied and developed several top-down and bottom-up production methods to overcome this challenge. Nonetheless, nowadays the electrochemical exfoliation^12^ (anodic or cathodic), the graphene oxide route,^13^ and the direct liquid-phase exfoliation method,^14^ are the most promissory methodologies^15^ to produce single- or few-layered graphene nanosheets in bulk quantities.

Specifically, the electrochemical exfoliation (also referred to as electrolytic exfoliation) has become an attractive methodology by its operative simplicity, easy scalability, and versatility to use a wide variety of electrolytes to get exfoliated graphene nanosheets with high yields.^16,17^ The exfoliation occurs when the ions from the electrolyte are intercalated between graphite layers in the graphite electrode, during the feed of an electrical current in an electrolytic cell. As a result, the interlaminar distance is increased, leading to exfoliated graphene layers with a high yield. In particular, for electrochemical exfoliation under cathodic conditions, the graphite electrode acts as a cathode and the cations intercalating from the electrolyte can be molten salts, salts in organic solvents, ionic liquids, etc.^18−20^ In contrast, anodic electrochemical exfoliation occurs when the graphite works as the anode, and anions from the aqueous electrolyte intercalate in the anode. It is worth noting that anodic exfoliation is considered a greener and environmentally respectful process, due to the use of water as the electrolyte which leads to a high yield of exfoliated layers; nevertheless, it can produce oxygen-centered radicals from the anodic oxidation of water that attack the exfoliated layers.^16^

On the other hand, the chemical surface modification of graphene prevents the restacking of the exfoliated layers and can potentially improve their electrochemical performance,^21,22^ since the new functional groups promote localized effects on the graphene layer, thus enhancing their physicochemical properties. In particular, several heteroatoms, including N, P, S, and B, have been incorporated into the graphene lattice^23−26^ (doping), and nitrogen-, sulfur-, and phosphorus-based functional groups (i.e., -NO_*x*_, -SO_*x*_, and -PO_*x*_) have been used to chemically functionalize the graphene nanosheets.^16,17,27,28^ Among them, phosphorus-based functional groups are highly desirable for electrochemical energy-storage devices since they possess an excellent electron-donating ability improving the band gap of the functionalized layers. In this sense, many methodologies to functionalize or dope graphene nanosheets with phosphorus groups have been reported; however, they are usually time-consuming or multistep processes that use expensive reagents and high-temperature post-treatments, or involve processes at high pressures.^23−26,29,30^

In this context, anodic exfoliation appears potentially as a powerful, affordable, and greener technique to produce exfoliated and phosphorus-containing nanosheets in one-step using mild reaction conditions without complex post-treatments, as evidenced by recent studies.^31^ Nonetheless, studies focused on electrochemical energy storage are scarce.^16,17,32^ Here, graphene nanosheets electrochemically exfoliated from graphite foil and phosphate-functionalized (denoted as phosphate-functionalized graphene (PFG)) were obtained in an expeditious and single-step process via anodic exfoliation at room temperature. The underlying exfoliation/functionalization mechanisms by electrochemical exfoliation of graphite using phosphoric acid (H_3_PO_4_) or sodium phosphate (Na_3_PO_4_) are proposed. Three concentrations of H_3_PO_4_ (0.25, 0.5, and 1 M) and Na_3_PO_4_ (0.05, 0.1, and 0.25 M), both as the phosphate sources and the electrolytes in an aqueous solution, were studied. On the other hand, regardless of the phosphate source (PFG-acid or PFG-salt), as the electrolyte molar concentration decreased, nanosheets with higher phosphate groups were obtained. Thus, PFG-acid at 0.25 M and PFG-salt at 0.05 M exhibited the highest phosphorus contents (2.2 and 1.4 at. %, respectively), and its specific capacitance was investigated with three- and two-electrode cells, achieving a competitive performance as a more sustainable and greener energy-storage device.

## Experimental Section

### Materials

A 500 μm-thick high-purity graphite foil (Papyex I980) was purchased from Mersen. Phosphoric acid (H_3_PO_4_, 85% w/w aqueous solution, ACS grade), and sodium phosphate dodecahydrate (Na_3_PO_4_·12H_2_O, 97%) were purchased from Alfa Aesar, and sulfuric acid (1 M H_2_SO_4_) was purchased from VWR Chemicals. Deionized water (DI H_2_O, resistivity: 18.2 MΩ·cm) was used throughout the experiments.

### Instrumentation

PFG nanosheets were dispersed in DI H_2_O and deposited by drop-casting onto preheated (50–60 °C) circular stainless-steel sample-holders (12 mm in diameter) for analysis by Raman spectroscopy and X-ray photoelectron spectroscopy (XPS). UV–vis absorption spectra of aqueous PFG dispersions were obtained with a double-beam Heλios α spectrophotometer (Thermo Spectronic). Raman analysis was carried out by using a Horiba Jobin-Yvon LabRam instrument at an incident laser power of 2.5 mW and a laser wavelength of 532 nm. The XPS measurements were accomplished on a SPECS apparatus at 10^–7^ Pa with a nonmonochromatic Mg Kα X-ray source (11.81 kV, 100 W). The recorded spectra were curve-fitted using the AAnalyzer software, and a Shirley-Sherwood background function and Voigt curve type were used. The thermogravimetric analysis was carried out in a TA Instruments SDT Q600 apparatus, heating the samples from room temperature to 900 °C at 10 °C/min under ultra-high-purity nitrogen flow (100 mL/min) and using platinum crucibles of 90 μL as the sampler container. Scanning electron microscopy (SEM) and energy-dispersive X-ray spectroscopy (EDS) analyses were performed using a Quanta FEG 650 apparatus from FEI Company. The samples were supported onto metallic holders with carbon adhesive tape and analyzed at a 20–25 kV voltage. Atomic force microscopy (AFM) imaging was performed in a Nanoscope IIIa Multimode system in the tapping mode of operation and using rectangular silicon cantilevers with resonance frequencies of 250–300 kHz and nominal spring constants of 40 N m^–1^. Specimens for AFM were prepared by drop-casting small volumes (∼20 μL) of an aqueous dispersion of the graphene sample (∼0.01 mg mL^–1^) onto freshly cleaved highly oriented pyrolytic graphite (HOPG) substrates the were preheated to 50–60 °C. Transmission electron microscopy (TEM) and selected area electron diffraction (SAED) measurements were carried out using a JEOL JEM 2100F microscope at an acceleration voltage of 200 kV on specimens prepared by drop-casting 20 μL of an aqueous dispersion of the sample (0.01 mg·mL^–1^) onto a copper grid (200 square mesh) covered with a lacey carbon film and allowing them to dry at room temperature. A graphene slurry was immersed in liquid nitrogen (−176 °C) and freeze-dried for 24 h using a Telstar Cryodos apparatus. The N_2_ adsorption isotherms were recorded at −196 °C in an ASAP 2420 volumetric apparatus (Micromeritics), after degassing the resulting powder overnight under vacuum at 110 °C. The specific surface areas were obtained from the adsorption branch of the N_2_ isotherms by the standard Brunauer–Emmett–Teller (BET) method in the relative pressure range 0.05–0.35. PFG films were prepared by vacuum-filtering aqueous dispersions of the corresponding sample through PTFE membrane filter. The electrical conductivity of the PFG films was determined through measurement of their resistance with a Fluke 45 digital multimeter. To this end, the films were cut into rectangular strips about 10 × 25 mm large and their thickness was measured with a thickness gauge.

### PFG via Anodic Exfoliation

The anodic exfoliation was carried out with a two-electrode configuration, using graphite foil (5 cm ×3 cm × 500 μm) as the anode and platinum foil (2.5 cm × 2.5 cm × 25 μm) as the cathode. Both electrodes were located in a face-to-face parallel position at 2 cm from each other. The electrodes were immersed in 80 mL of an aqueous solution of H_3_PO_4_ or Na_3_PO_4_ at a specific concentration (see Table 1). Then, a bias voltage of 10 V was applied to the anode for 40 min using an Agilent 6614C DC power supply. Upon applying the voltage, the graphite foil anode was seen to swell and break down into tiny particles that were released to the electrolytic solution. The resulting dispersed solid was collected and filtered using a cellulose membrane (4 μm of pore size) to obtain a black slurry, which was purified by redispersion (assisted by a brief ultrasonic treatment) in DI H_2_O/centrifugation, at 200*g* for 20 min to obtain an aqueous colloidal suspension, several times until the centrifugated reached a pH value of ∼6 or a resistivity of ∼2 MΩ·cm when H_3_PO_4_ or Na_3_PO_4_ were used as the electrolyte, respectively. Finally, the slurry was dried at 60 °C for 12 h under a vacuum.

**Table 1:** Characteristics of the Phosphate-Functionalized Graphene-Based Materials Obtained by Aqueous Anodic Exfoliation of Graphite Foil with Different Phosphate Source Concentrations (Acid and Salt)^a^

	[M]	yield		[phosphorus] (at.-%)	O/C with PO_4_^3–^	O/C without PO_4_^3–^	*I*_D_/*I*_G_
		(mg/min)	(wt-%)				
H_3_PO_4_	0.25	3.7	30	2.2	0.28	0.17	1.12
	0.50	7.8	62	1.4	0.21	0.14	1.07
	1.00	5.3	37	1.1	0.14	0.09	0.96
Na_3_PO_4_	0.05	3	26	1.4	0.22	0.15	0.86
	0.10	3.3	29	1.1	0.19	0.14	0.84
	0.25	5.2	46	0.4	0.15	0.13	0.66

a The phosphorus content and O/C ratios were obtained from XPS, and *I*_D_/*I*_G_ ratio from Raman analysis.

### Electrochemical Energy-Storage Measurements

The electrochemical energy storage experiments were performed in both three-electrode and two-electrode configurations with a Swagelok-type cell. In the three-electrode configuration PFG nanosheets were used as the working electrode (WE), commercial activated carbon fiber as the counter electrode (CE), and Ag/AgCl as the reference electrode (RE) in 1 M H_2_SO_4_ (as the electrolyte). On the other hand, in the two-electrode configuration the PFG nanosheets were used as both the WE and CE in the same electrolyte (1 M H_2_SO_4_). The graphene-based WE and CE were prepared by pressing a certain amount of dried PFG sample onto a circular piece of graphite foil (1 cm^2^), to a mass loading of around 1 mg cm^–2^. The carbon fiber-based CE was incorporated in paste form, which comprised activated carbon fiber as the active component, polytetrafluoroethylene as a binder, and carbon black as a conductive additive, in a weight ratio of 90:5:5, respectively. Two stacked nylon membrane filters (0.20 μm of pore size, Whatman) were used as the electrode separator. Before the cell was assembled, the WE and CE were individually soaked in 1 M H_2_SO_4_, and the assembled cell was vacuum-degassed. The energy storage performance measurements were carried out in a VSP potentiostat (Bio-Logic Science Instruments), recording cyclic voltammograms at different sweep rates (3–500 mV s^–1^) and galvanostatic charge–discharge curves at different potential current densities (0.1–20 A g^–1^), using an electrochemical window between −0.2 and 0.9 V vs Ag/AgCl for measurements in the three-electrode configuration and between 0 and 1.0–1.1 V in the two-electrode configuration. Electrochemical impedance spectroscopy (EIS) measurements were carried out for the fully discharged cell using a potential amplitude of 10 mV in a frequency range from 100 kHz to 0.1 Hz.

## Results and Discussion

### PFG: Synthesis, Characterization, and Mechanisms

**Figure 1:**
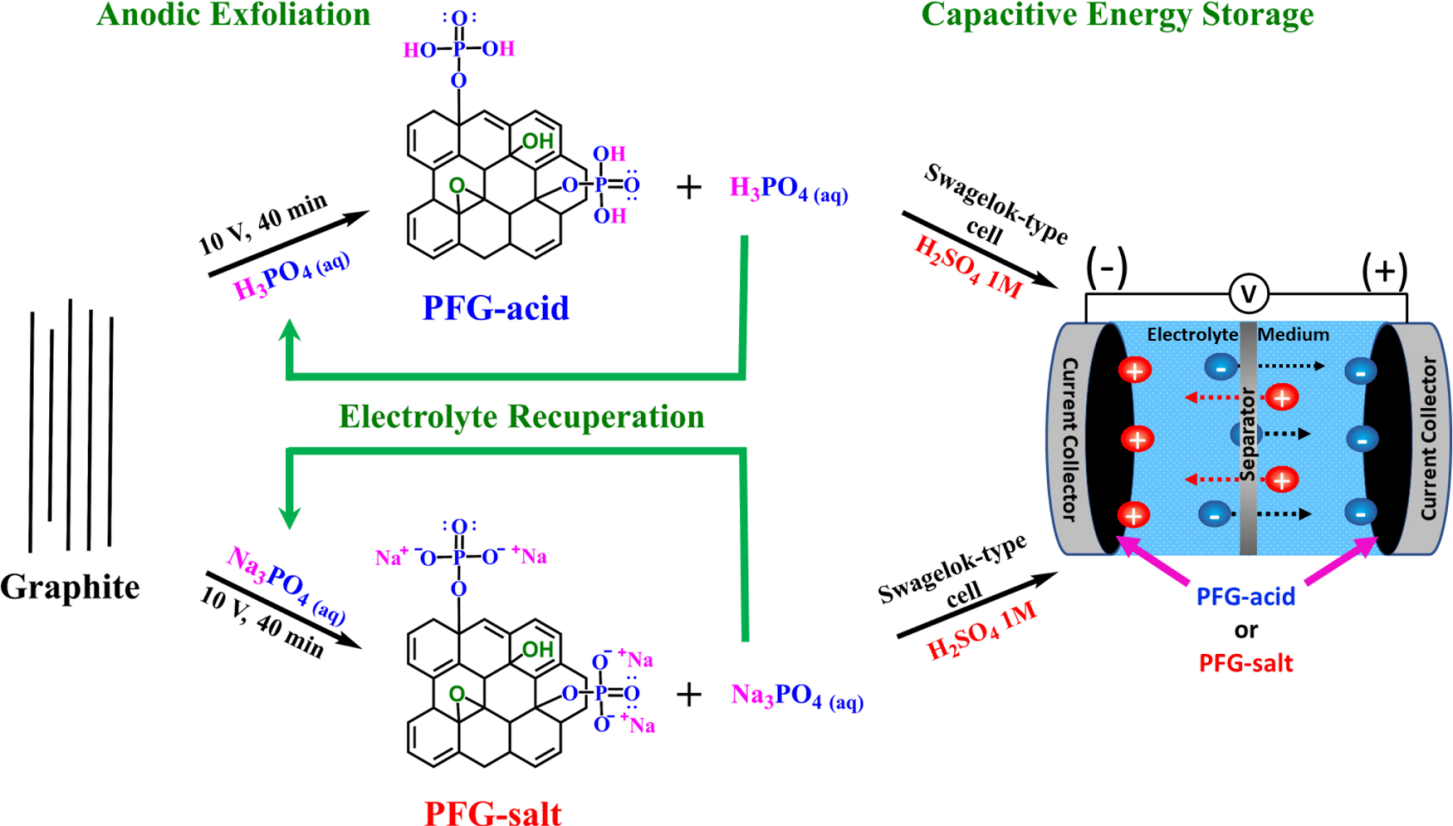
Schematic representation of a green process to achieve phosphate-functionalized graphene (PFG-acid or PFG-salt) via anodic exfoliation using H_3_PO_4_ or Na_3_PO_4_ as phosphate source/electrolyte and its use as an electrode material for capacitive energy storage.

Graphene nanosheets decorated with phosphate groups were readily obtained in a single step via anodic exfoliation at room temperature in a short time using aqueous solutions of H_3_PO_4_ or Na_3_PO_4_ as the electrolyte and phosphate source, as shown in Figure 1. Here, graphite foil was used as the graphene nanosheets precursor, which when used with the phosphate-based electrolytes gives PFG materials with outstanding and competitive energy-storage characteristics. In particular, the attractive preparation conditions (a fast and room-temperature process using mild chemicals) and the ability to reuse the electrolyte promotes a circular-synthesis process to achieve a sustainable and greener energy-storage device successfully.

A few works have made use of anodic exfoliation as a powerful tool to obtain phosphate-based functional groups on graphene nanosheets or reduced graphene, owing to the fact that this technique is simple, fast, environmentally friendly, and scalable. Lee et al.,^31^ concluded that using a graphite source with compact layer stacking (HOPG) and H_3_PO_4_ as the electrolyte, it was impossible to achieve phosphate-functionalized nanosheets by anodic exfoliation; nonetheless, Zu et al.^32^ achieved the phosphate functionalization via anodic exfoliation using a graphite rod and ammonium phosphate as the electrolyte. Moreover, Munuera et al.^16^ demonstrated that effective electrochemical exfoliation of graphite in different electrolytes is strongly dependent on the type of graphite source used (i.e., compact vs noncompact type of layer stacking in the graphite material). In the same way, we found that the use of graphite with noncompact stacking such as graphite foil, readily afforded the exfoliation and functionalization of the material via anodic exfoliation with a phosphate-based aqueous electrolyte. As is well known, the electrolyte concentration is also a critical factor in the anodic exfoliation process.^16^ For this reason, to understand the influence of this parameter on the chemical and morphological characteristics of the resulting PFG nanosheets and their energy-storage behavior, both electrolytes (H_3_PO_4_ and Na_3_PO_4_) were used at different concentrations.

Table 1 shows the concentrations of H_3_PO_4_ and Na_3_PO_4_ electrolytes tested here. We determined the concentration range where the exfoliation process can be successfully achieved for both electrolytes. As a general rule, when the electrolyte concentration is too low, no exfoliation process is attained; this phenomenon was observed for H_3_PO_4_ at 0.10 M and Na_3_PO_4_ at 0.01 M. On the other hand, if the electrolyte concentration is too high, a very fast expansion and detachment of graphite sheets from the anode takes place. As a result, the attainment of well-exfoliated graphene nanosheets is not possible, probably because the fast detachment of graphite fragments, leaves no time for an efficient layer-by-layer intercalation of the material to occur.^16^ This behavior was observed for H_3_PO_4_ at concentrations above 1 M. For the Na_3_PO_4_, its solubility in water is not possible at concentrations higher than 0.25 M, so this was the upper concentration limit for this electrolyte. It is also worth noting that increasing the electrolyte molar concentration promotes an increment in the yield and consequently an improvement in the solution conductivity.^33^ Thus, a good formation of intercalating aqueous ions and a better electrical exfoliation are obtained.

**Figure 2:**
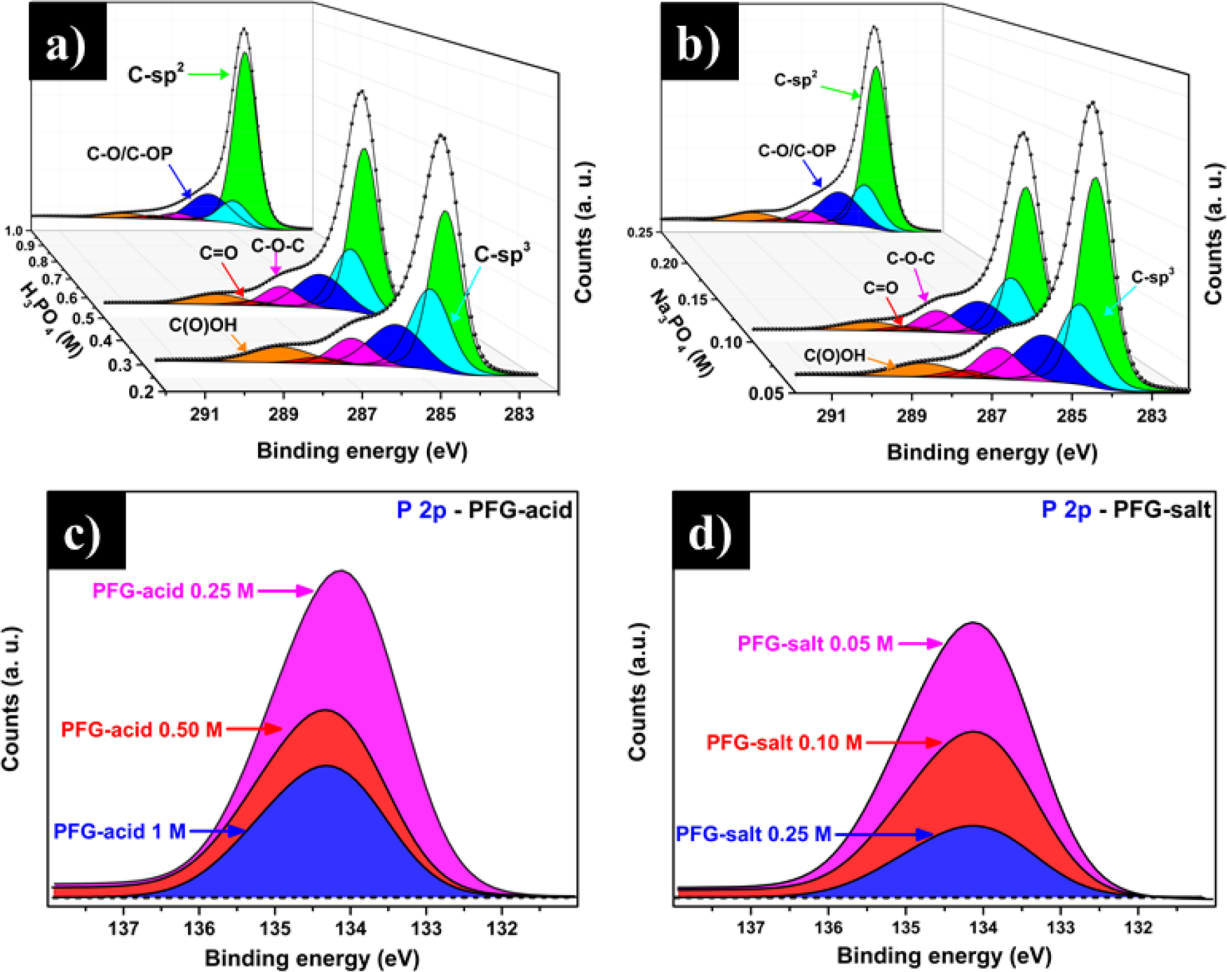
Chemical states characterization of C 1s and P 2p by high-resolution X-ray photoelectron spectroscopy (HR-XPS) corresponding to (a and c) PFG-acid, and (b and d) PFG-salt at different concentrations.

High-resolution XPS (HR-XPS) analysis of C 1s and P 2p corresponding to PFG-acid and PFG-salt at different concentrations were carried out to examine the new chemical states. Figure 2 shows the HR-XPS spectra of C 1s and P 2p corresponding to graphene exfoliated with both electrolytes. The C 1s curve was deconvoluted into six components, which were assigned to C-sp^2^ (C=C, 284.4 eV), C-sp^3^ (C–C, 284.8), C–O/C–OP (285.7 eV), epoxy (C–O–C, 286.7 eV), carbonyl (C=O, 287.7 eV), and carboxyl (288.7 eV, O–C=O).^27,34,35^ The composition values obtained by the curve-fitting are shown in Table S1 (Supporting Information). It is worth highlighting that regardless of the electrolyte type or concentration used to obtain PFG-acid or PFG-salt, the final materials possess oxygen-based functional groups. The O/C ratio without contribution from phosphate groups was calculated to determine the presence of the oxygen-based functional groups, which suggested an oxidized material with an oxygen content within the common range for anodically exfoliated graphene.^36,37^

In fact, the oxidation obtained is similar that of graphene oxide after being subjected to an efficient reduction process.^38^ Using H_3_PO_4_ or Na_3_PO_4_ as electrolytes at different concentrations results in exfoliated graphene with different O/C ratios, as shown in Table 1. As the electrolyte concentration is increased, the oxygen amount in the exfoliated graphene samples tends to decrease. This finding is consistent with the fact that, at high electrolyte concentrations, a faster exfoliation/detachment of graphene nanosheets at the anode takes place and thus their chances to get oxidized during the electrolytic process decrease as the residence time of the graphene layers at the anode also decrease.

Furthermore, to get a more comprehensive insight into the phosphate species chemically bonded to graphene layers, HR-XPS P 2p analysis was performed, as shown in Figure 2c,d. In this case, samples of PFG-acid and PFG-salt, regardless of the concentration used, displays only one component observed at ∼134 eV, corresponding to phosphate-based functional groups with the general structure O=P–(OR)_3_.^26,39^ This curve comprises the 2p_1/2_ and 2p_3/2_ contributions with a gap of 0.95 eV of separation and a ratio of 2:1 (2p_3/2_: 2p_1/2_), which is typically observed for phosphate compounds.^39^ It is worth noting that all characterizations performed here were carried out after a thorough purification process of all PFG samples to remove remnants of electrolyte as well as free, nonbonded chemical species. Therefore, it is reasonable to assume that the phosphate groups observed into graphene samples by XPS are those that are chemically bonded to the graphene nanosheets.

Nonetheless, the use of lower molar concentrations of electrolytes leads to PFG nanosheets richer in phosphorus. Also, as revealed in Figure 2c, the PFG-acid sample at 0.25 M possesses the highest phosphorus content compared to those concentrations of PFG-acid at 0.50 and 1 M. The phosphorus content (at. %) in all samples is summarized in Table 1. In the same way, using the PFG-salt at a lower molar concentration (0.05 M, Figure 2d) exhibited the highest phosphorus content, compared with higher salt concentrations used (0.10 and 0.25 M). Notice that the PFG material derived from 0.25 M H_3_PO_4_ possesses the highest phosphorus content, and indeed it has the highest oxygen concentration (see Table S1, Supporting Information). The oxidized species reduction suggests that when the electrolyte concentration is increased, it promotes a faster intercalation, minimizing the graphene oxidation. Thus, using low electrolyte concentration (0.25 M H_3_PO_4_ and 0.05 M Na_3_PO_4_) allows a better intercalation and aqueous ions diffusion. As a result, a slower detachment and layer-by-layer exfoliation was obtained. For this reason, the PFG nanosheets produced using low electrolyte concentrations were exposed to oxidizing conditions for longer times, and as such they were more functionalized with oxygen- and phosphate-based functional groups, as shown in Table 1.

**Figure 3:**
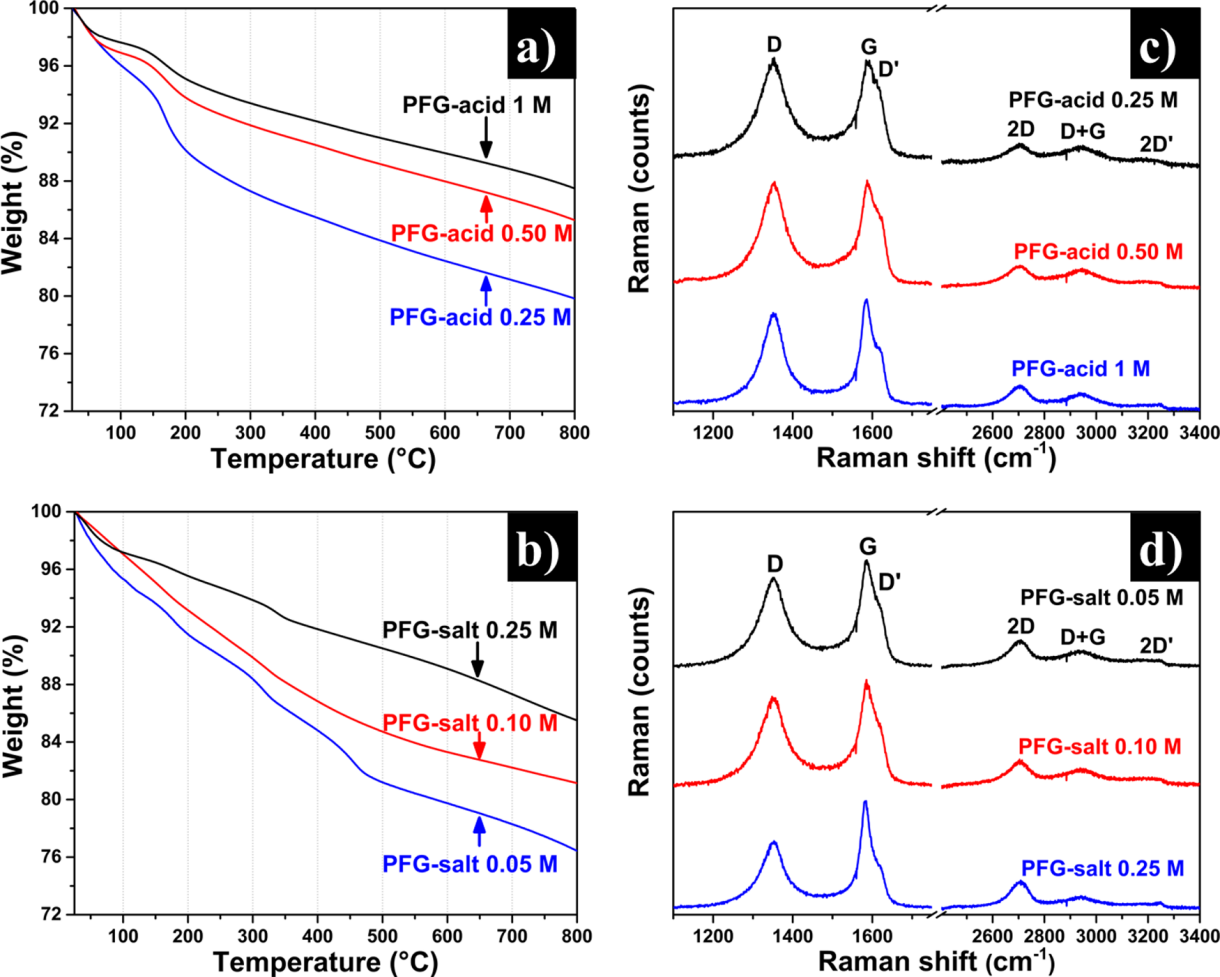
(a, b) Thermogravimetric analysis and (c, d) Raman spectroscopy corresponding to PFG-acid (H_3_PO_4_) and PFG-salt (Na_3_PO_4_) at different concentrations.

On the other hand, the thermostability and the structural information on the exfoliated material were obtained by thermogravimetric analysis (TGA) and Raman spectroscopy, respectively, as shown in Figure 3. H_3_PO_4_ or Na_3_PO_4_ as electrolytes at different concentrations produce PFG materials with different thermostability and hygroscopic properties (weight loss around 100 °C), as shown in Figure 3a,b.

It is well known that chemisorbed water molecules and many oxygen-containing functional groups in highly oxidized graphenes can be removed at temperatures around 200 °C.^40^ In particular, desorption of chemisorbed water around this temperature can be reasonably related to water molecules associated to the phosphate groups^23^ and decomposition of nearby-lying oxygen groups located on the basal surface of graphene to give CO and CO_2_ also takes place at temperatures around 150–250 °C.^41^ Decomposition of oxygen groups bound to edges, rather than basal surfaces, leads to CO/CO_2_ evolution and hence to weight losses across the whole temperature range above ∼300 °C. When the electrolyte concentration was increased, a decrement in the weight loss, both at around 200 °C and at higher temperatures, was generally observed for the PFG-acid and PFG-salt materials. This result was consistent with the lower extent of functionalization (oxidation and phosphatation) of the graphene nanosheets with increasing electrolyte concentration that the XPS measurements revealed. Moreover, the presence of oxygen-containing and phosphate functional groups favors the interaction of the graphene materials with the atmospheric water molecules, implying that the amount of absorbed water will increase; this phenomenon is observed in both PFG (acid and salt) materials. The wettability is an essential property in the materials applied in the electrochemical energy storage since this property promotes a better interaction of the aqueous ions with the electrode surface.

The presence of defects of the PFG nanosheets was evaluated by Raman spectroscopy, as shown in Figure 3c,d. In all cases, the three typical Raman bands of graphitic/graphene materials, namely, the D, G, and D′ bands, located at ∼1350, 1580, and 1620 cm^–1^, respectively,^16,37^ appeared in the first-order region of the spectra. Also, the 2D band was seen in the second-order region of the spectra at ∼2700 cm^–1^. The G band corresponds to C–C bond-stretching vibrations in the sp^2^ lattice graphite/graphene materials, whereas D and D′ are defect-related bands.^42^ The intensity ratio between D and G bands (*I*_D_/*I*_G_ ratio) can be used as a quantitative measure of the structural defects and imperfections present in the lattice.^42^ As is well known, the intensity of the 2D band is also correlated with the amount of defects in the graphene lattice:^43^ a low intensity of the 2D band implies a high concentration of defects.

More specifically, the increase in electrolyte concentration produces nanosheets with less structural disorder. This result is related to the amount of oxidized carbon species, as can be observed in the O/C ratios (Table 1), which means more structural defects. As a consequence, a decrease in the *I*_D_/*I*_G_ ratio and an increment in the 2D band intensity were observed, indicating moderately oxidized graphene materials. As revealed by the *I*_D_/*I*_G_ ratio and the 2D band, the use of H_3_PO_4_ as electrolyte leads to PFG materials having slightly higher structural disorder than those observed using Na_3_PO_4_-derivated materials. These results are in accordance with the O/C ratio behavior. Additionally, the PFG materials were analyzed by UV–vis spectroscopy. It can be observed that the peak for the different PFG-acid or PFG-salt samples is located between 260 and 270 nm, as shown in Figure S1a,b (Supporting Information). This absorption peak corresponds to π → π* transitions from electronically conjugated domains in carbon materials,^16,21^ and its position at 260–270 nm is associated to graphitic/graphene materials with low to moderate oxidation levels.^37^ These results are thus consistent with the O/C ratios obtained by XPS for the present graphene samples (Table 1).

**Figure 4:**
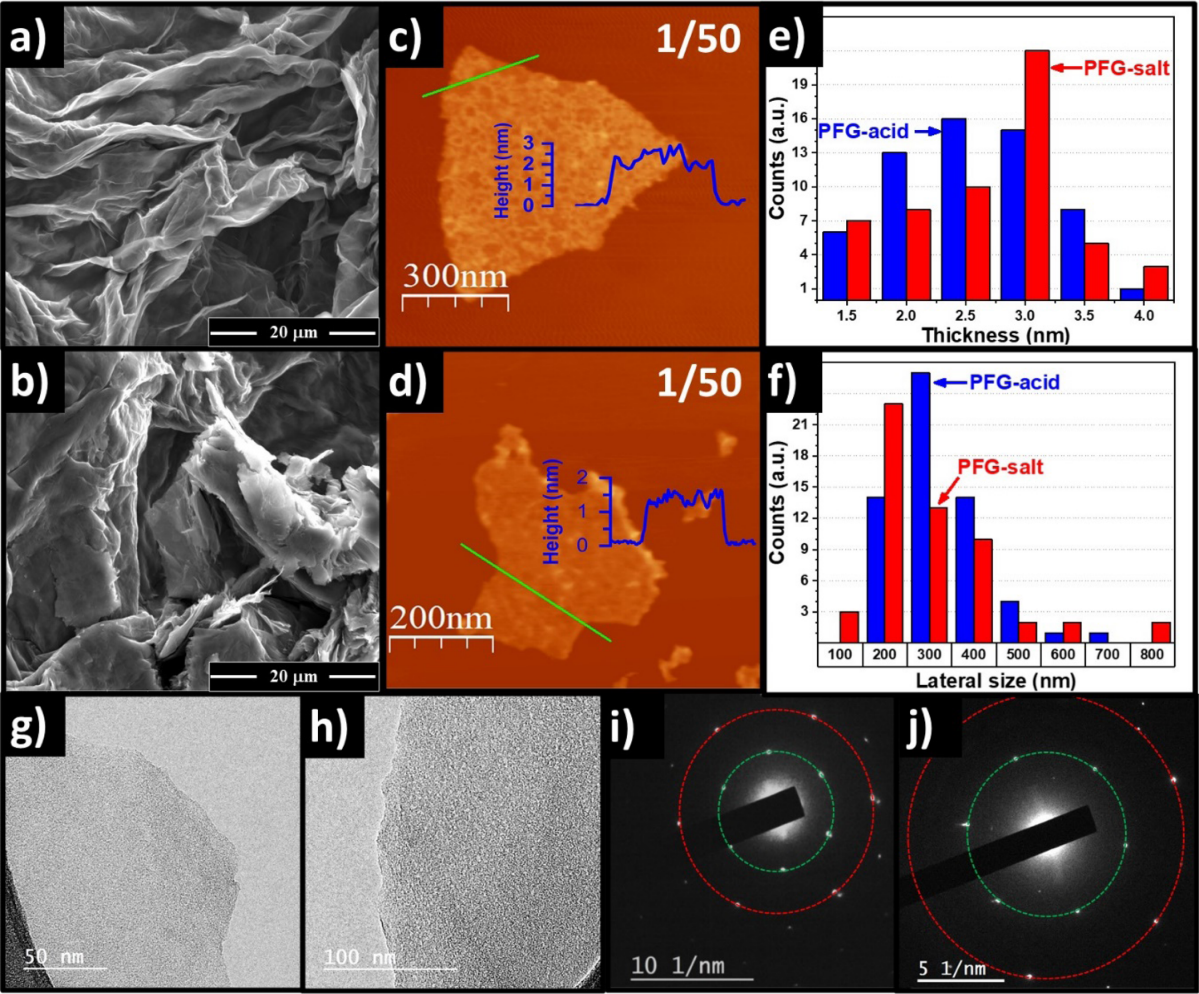
Scanning electron microscopy (SEM) images at higher magnifications corresponding to (a) PFG-acid and (b) PFG-salt. Atomic force microscopy (AFM) images of (c) PFG-acid and (d) PFG-salt. AFM statistical analysis of 50 nanosheets for (e) thickness and (f) lateral size distribution. Transmission electron microscopy (TEM) images of (g) PFG-acid and (h) PFG-salt, and their corresponding electron diffraction patterns for (i) PFG-acid and (j) PFG-salt.

To study the morphology of the PFG materials, SEM coupled with EDS, AFM, TEM, and SAED analyses were carried out, as shown in Figure 4. The samples with the highest phosphorus content, PFG-acid (0.25 M) and PFG-salt (0.05 M) at low resolution are shown in Figure 4a,b, respectively, evidencing corrugated and distorted morphologies. Such morphologies originated during the anodic exfoliation process where the intercalated ions and water molecules induce an expansion of the interlayer space of the graphite anode, leading to the corrugation and distortion of the expanded nanosheets. The PFG-acid material appeared to have higher exfoliation level, in which the PFG-acid sample possesses more corrugated layers, and its PFG-salt counterpart possesses thicker flakes in a more stacked configuration.

As shown in the AFM images (Figure 4c,d) the lateral size of the PFG ranges from ∼200 to ∼700 nm. Moreover, as shown in Figure 4e,f, the histograms indicated that PFG possess a thickness less than 4 nm, which suggested that the materials were well exfoliated, as well as confirmed that PFG-acid nanosheets are generally somewhat thinner and larger than PFG-salt nanosheets. This finding is consistent with the fact that PFG-acid possess a higher BET specific surface area (110 m^2^ g^–1^) compared with the PFG-salt (22 m^2^ g^–1^), as shown in Figure S2 (Supporting Information), which is related to the exfoliation level. The ultrasonic/centrifugation treatment can be expected to select (i.e., retain in the supernatant) those nanosheets with similar lateral sizes (typically several hundreds of nanometers or a few micrometers at most) and also with a surface chemistry that has a high affinity toward water (nanosheets functionalized with oxygen and phosphate groups), eliminating those (hydrophobic) nanosheets that are not functionalized, if the latter are really present in the particles. The introduction of a small amount of functional groups to graphene nanosheets, it is possible to increase the water dispersion stability without disturbing the inherent properties.^28^ On the other hand, it is very difficult to actually determine whether different particles detached from the graphite anode possess somewhat different surface chemistries. However, this is possibly not the case: the graphite foil electrode is rather compact and should not be easy to be infiltrated by the aqueous electrolyte. This means that, at any given time, only the graphite microparticles that are strictly located on the surface of the foil will be expanded and finally detached from the electrode. Only after this happens, new deeper-lying microparticles will become exposed to the electrolyte, so that they can be expanded by the electrolytic treatment and detached. Such a course of action implies that the time any given graphite microparticle is actually subjected to the electrolytic treatment before detaching will be more or less similar for all the particles, and therefore their overall characteristics (e.g., surface/interfacial chemistry, degree of expansion) should also be similar.

Furthermore, TEM and electron diffraction were used to evaluate the crystalline quality of the as-prepared PFG sheets. A careful examination on the edge structure confirmed that the PFG-salt and PFG-acid materials were well exfoliated and appeared as continuous objects with uniform thickness over the area, as shown in Figure 4g,h. Furthermore, the electron diffraction patterns for PFG-acid (Figure 4i) and PFG-salt (Figure 4j) showed one set of hexagonal patterns expected for highly ordered graphite/graphene structures.^44^ Specifically, the green and red dotted circles in the reciprocal space corresponded to electron diffraction by crystallographic planes with separations of 2.13 and 1.23 Å, respectively, which could be assigned to the {10–10} and {11–20} planes. These results confirmed that PFG-acid and PFG-salt possessed substantial crystallinity in their basal planes,^45^ even though they were functionalized to a significant extent with oxygen and phosphate groups. It is worth highlighting that PFG possesses a competitive electrical conductivity with other materials obtained via anodic exfoliation,^37^ in which the PFG-acid and PFG-salt yielded values of 8300 and 6600 S m^–1^, respectively, which is related to the substantial crystallinity previously observed.

In addition, the presence of the phosphate-based functional groups in the nanosheets was evidenced by EDS mapping of phosphorus (P Ka1). As evidenced by the corresponding EDS elemental maps, phosphorus (Figure S3d,h, Supporting Information) was uniformly incorporated throughout the nanosheets. The EDS-derived atomic percentage corresponding to phosphorus was 1.5 and 1.6 at. % for PFG-acid and PFG-salt, respectively, which was comparable to the values determined by XPS (2.2 and 1.4 at. %). Oxygen was also uniformly distributed throughout the graphene samples, as expected (Figure S3c,g, Supporting Information).

**Figure 5:**
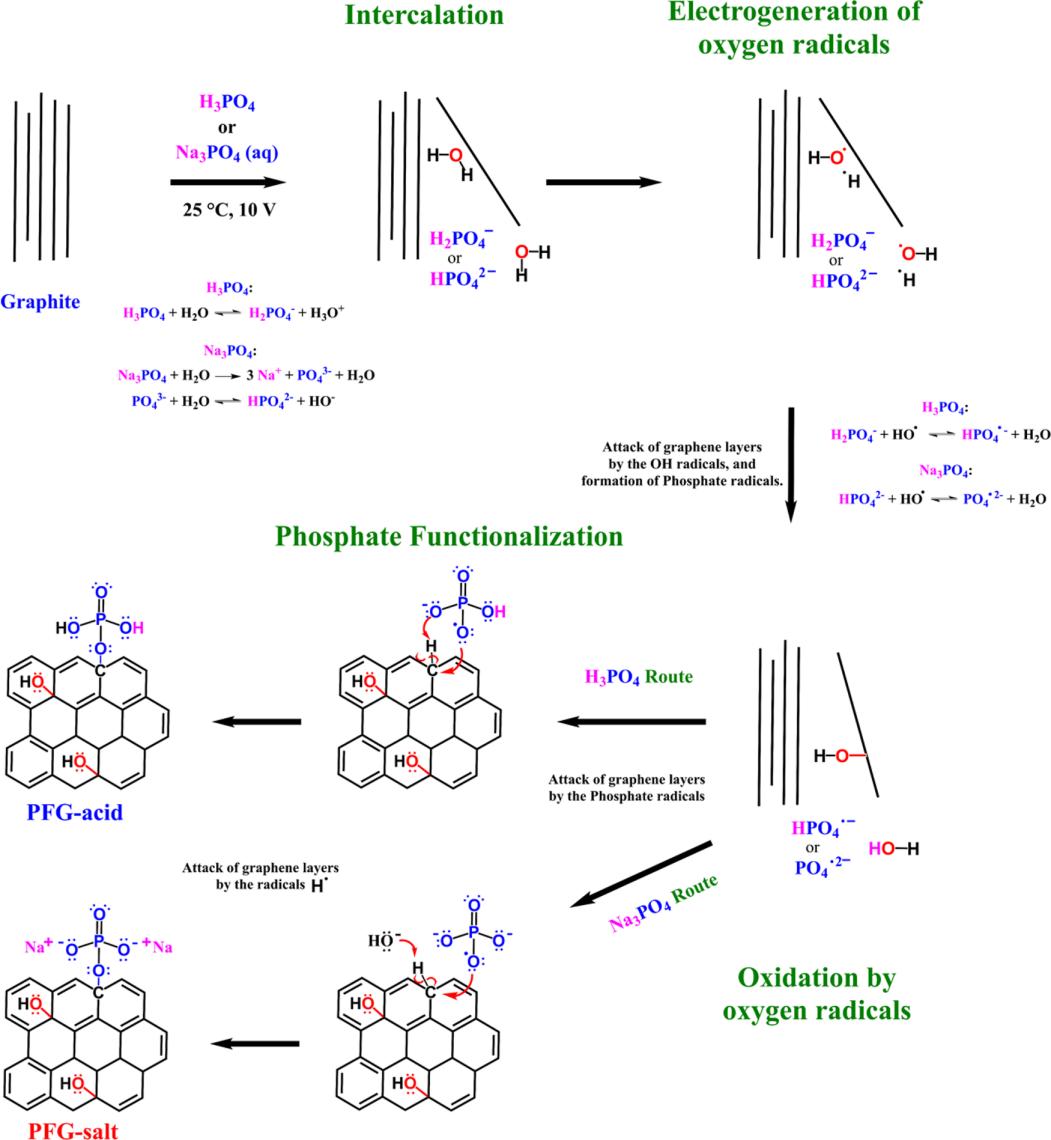
Functionalization mechanism of the sequentially one-step synthesis process corresponding to phosphate functionalized graphene (PFG) using acid (H_3_PO_4_) or salt (Na_3_PO_4_) source via anodic exfoliation.

The addition of phosphorus-based functional groups to graphene nanosheets is potentially attractive due to their electron-donating ability.^39^ Here, we propose an anodic exfoliation/functionalization mechanism of graphite to give graphene nanosheets decorated with phosphate groups, as shown in Figure 5. The synthesis is accomplished in a one-pot procedure and is proposed to comprise four main processes. The first is *intercalation:* The graphite foil (anode) undergoes exfoliation by intercalation of ions (e.g., phosphate aqueous ions) with cointeraction of water molecules from an aqueous electrolyte solution.^16^ Specifically, the H_3_PO_4_ and Na_3_PO_4_ electrolytes possess different species; X_2_PO_4_^–^, XPO_4_^2–^, and PO_4_^3–^ (X = H, Na). Since H_3_PO_4_ and Na_3_PO_4_ are a weak acid and weak base, respectively, the predominant species are and H_2_PO_4_^–^, and PO_4_^–3^ and HPO_4_^2–^. The second process is *electrogeneration of oxygen radicals*: the use of a large anodic overpotential (e.g., 10 V); active intermediates from water decomposition (e.g., •OH) are obtained at the graphite interface.^46^ These radical oxygen species tend to oxidize the subsequent exfoliated graphene nanosheets. On the other hand, we know from previous electrochemical exfoliation experiments carried out in our laboratory with different aqueous electrolytes that working at bias voltages of 8 or even 6 V still affords exfoliation of the graphite anode, although at somewhat lower yields, but exfoliation becomes inefficient at even lower voltages. Therefore, a bias voltage of 10 V appears as a reasonable compromise between efficiency of exfoliation and energy consumption. The third process is *oxidation by radicals:* Radical species oxidize the graphene layers^16^ and increase the aromatic ring reactivity.^47^ More specifically, the -OH substituent possess a high resonance donator effect, favoring further functionalization in *ortho-* or *para-*substitutions.^47,48^ Furthermore, the presence of •OH radicals can be expected to induce the formation of phosphate radical species (from phosphate anions), thus increasing the reactivity of this species.^49,50^ The fourth process is *phosphate functionalization.* The radical phosphate functional groups react with carbon atoms from the graphene in an addition reaction.^48,50,51^*Para-*substitution would be favored since the phosphate radicals possess a significant volume and a steric hindrance phenomenon could be present with -OH substituent. The functionalization routes for both materials are slightly different, since the H_3_PO_4_ electrolyte route is an acid medium and the Na_3_PO_4_ electrolyte a basic medium. The formation of one C–O–P is supported by the atomic ratio Na/P (2:1) obtained by EDX.

### PFG as a Capacitive Energy-Storage Device

Previous studies on PFG materials as an electrode for electrochemical capacitors demonstrated enhanced energy-storage performance owing to the presence of the phosphate functional group.^25,39^ The present PFG materials were explored as an electrochemical energy-storage electrode in aqueous 1 M H_2_SO_4_ electrolyte using both three- and two-electrode configurations. The characterization by cyclic voltammetry(CV) curves and galvanostatic charge–discharge (GCD) profiles at different sweep rates and current densities, as well as by EIS, was carried out for the different electrolyte concentrations in a three-electrode configuration. The recorded CV curves are shown in Figure S4 (Supporting Information) and exhibited approximately rectangular shapes for a voltage window of 1.1 V (−0.2 to 0.9 V vs Ag/AgCl, limited by the electrolytic decomposition of water), which is indicative of a charge storage behavior dominated by capacitive processes.^35,39^ All the materials exhibited pseudocapacitive (Faradaic) reactions in the form of a pair of redox peaks in the 0.3–0.6 V range (vs Ag/AgCl), attributed to reversible C=O/C–O^–^ reactions in quinones and P=O/P–O^–^ in phosphate groups on the carbon surface.^25^ Remarkably, the samples with higher capacitance were the samples with more functional groups. There are two main reasons for this result, owing to the increase of functional groups: (i) an increment in the hydrophilicity of the nanosheets and (ii) increased redox pseudocapacitance. This phenomenon was observed for PFG nanosheets obtained with both phosphate sources (acid or salt).

**Figure 6:**
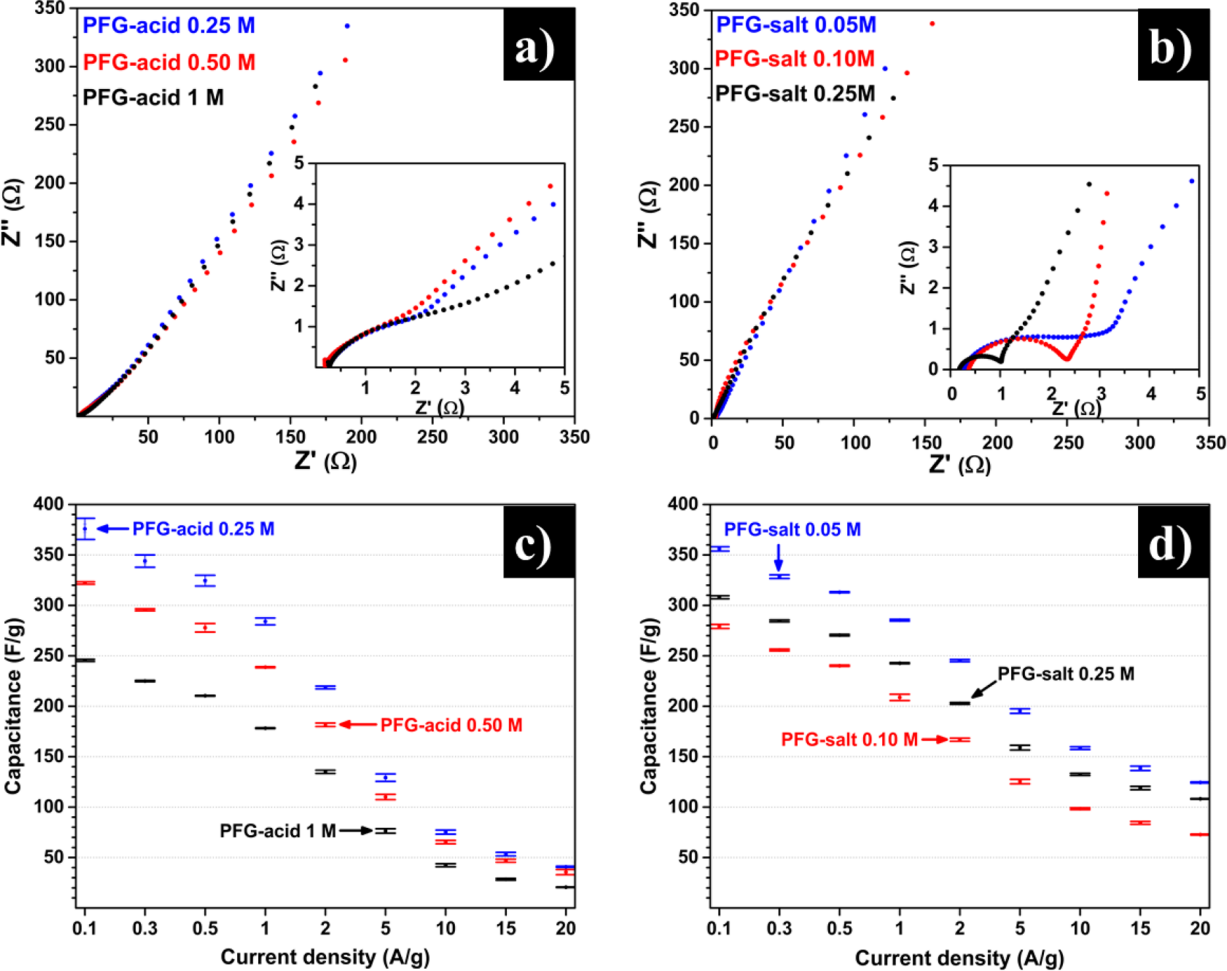
Nyquist plots (a, b) and gravimetric capacitance vs current density plots (c, d) corresponding to PFG samples obtained via anodic exfoliation at different electrolyte concentrations: (a, c) PFG-acid (H_3_PO_4_) and (b, d) PFG-salt (Na_3_PO_4_).

Similarly, EIS measurements can be used to explore the resistance and capacitive properties of the PFG-acid and PFG-salt samples from their corresponding Nyquist plots,^26^ as shown in Figure 6a,b, respectively. Figure 6a inset illustrates identical curves regarding their semicircular shape but with different resistance values (semicircle radius), corresponding to PFG-acid at different concentrations. Figure 6b inset shows a notorious difference in semicircle radius, according to the electrolyte concentration variation. Notably, using the electrolyte concentrations proposed herein, it is possible to modulate the charge-transfer resistance property. The PFG-salt samples showed shorter Warburg regions compared with their acid counterparts, which meant that the former possessed a better diffusion ability of the aqueous ions. This finding could be related to the characteristic solvation property of the new functional group C–O–PO_3_Na_2_ (PFG-salt), which permits its use as an efficient auxiliary dispersant in aqueous solutions.^52^ In the same way, this property allows water molecules and electrolyte ions to diffuse easier throughout the active material, compared with the C–O–PO_3_H_2_ functional groups (PFG-acid). This observation is consistent with the Bode plot analysis (Figure S5, Supporting Information), which shows a change in the capacitive process at medium and low frequencies (capacitive and resistance behavior region) according to the content of phosphorus-centered functional groups.

GCD profiles from the three-electrode cell within a potential window of 1.1 V are shown in Figure S6 (Supporting Information). All profiles exhibited an essentially triangular shape, which is characteristic of a dominantly capacitive process.^53^ Nonetheless, they showed a very slight slope change at 0.4–0.5 V (vs Ag/AgCl), associated with the above-mentioned pseudocapacitive reactions. These results are in good agreement with the observed in CV curves. The specific capacitance values derived from the GCD profiles for the different graphene samples at different current density are provided in Figure 6c,d. Figure 6c shows the capacitance values at three concentrations of PFG-acid, confirming that the sample with the most functional groups (PFG-acid at 0.25 M) is the material with more specific capacitance (375 F/g). This result showcases that higher number of functional groups imply more reactive sites.^54^ Moreover, in the PFG-salt samples, the material with a higher functional groups content is the sample that exhibits a higher specific capacitance (356 F/g), in contrast to materials obtained using other concentrations (Figure 6b).

The PFG-salt and PFG-acid nanosheets possess similar O/C ratios, but the PFG-acid possess higher phosphorus content and exfoliation level, which determined the higher specific surface area and conductivity values. Therefore, the difference in the capacitance loss at high current densities is mainly attributed to the different ion diffusion rates throughout the electrode material. This phenomenon is observed in the formation of diffusion layer (denominated as Warburg zone), where the PFG-salt nanosheets possessed a shorter process (1–2 Ω), compared with PFG-acid, as shown in the insets of Figure 6a,b.

The capacitive behavior of the PFG-acid and PFG-salt is competitive compared with other products (Table 2), whose synthesis conditions include high temperatures (from 180 up to 900 °C), high pressure reactors, and long reaction times (up to 12 h). Recently, Tanguy et al.^39^ developed a graphene-based material with a high phosphorus content (3.2 at. %); nonetheless, the energy storage capacitive was 260 F/cm^3^.

**Table 2:** Energy Storage Capacitive Values Corresponding to Different Carbon Sources Functionalized with Phosphorus-Centered Functional Groups

reference	synthesis conditions	electrolyte	capacitance (F/g)
Wang et al.^55^	GO with H_3_PO_4_ annealed at 800 °C/2 h. Phosphorous = 3.4 at. %	6 M KOH	130
Karthika et al.^24^	RGO with H_3_PO_4_ annealed at 200 °C until evaporation	1 M H_2_SO_4_	368
Wang et al.^35^	nitrogen-doped GO with H_3_PO_4_ annealed (twice) at 500 °C/4 h and 900 °C/1 h. Phosphorus = 1.6 at. %	6 M KOH	245
Thirumal et al.^56^	graphite rod exfoliated with H_3_PO_4_ and annealed at 400 °C/4 h. Phosphorus = 0.68 at. %	0.5 M H_2_SO_4_	290
Bi et al.^26^	reduced graphene with H_3_PO_4_ and annealed at 800 °C/2 h and 1000 °C/2 h. Phosphorus = 2.9 at. %	6 M KOH	108
Fan et al.^38^	GO with (a) phytic acid, (b) H_3_PO_4_, and (c) KH_2_PO_4_ hydrothermal process at 170 °C/12 h. Phosphorus = (a) 0.9, (b)1.3, and (c) 2.3 at. %	1 M H_2_SO_4_	(a) 388(b) 351(c) 318
Yang et al.^34^	polyimide solution with ammonium polyphosphate with thermal at 300 °C and laser. Phosphorus = 2.6 at. %	3 M KOH	206
Tanguy et al.^39^	GO with H_3_PO_4_ hydrothermal at 180 °C/5 h. Phosphorus = 3.2 at. %	1 M H_2_SO_4_	260 F/cm^3^
Suresh et al.^23^	RGO with Na_3_PO_4_ supercritical fluid at 400 °C/1 h. Phosphorus = 1.5 at. %	1 M H_2_SO_4_	518
this work	Grafoil anodic exfoliated with (a) Na_3_PO_4_ and (b) H_3_PO_4_ at room temperature for 40 min. Phosphorus = (a) 1.4 and (b) 2.2 at. %	1 M H_2_SO_4_	(a) 356(b) 375

In this work, we have obtained graphene materials with substantial phosphorus content in the form of phosphate groups via a greener, one-step synthesis strategy that is attractive compared to other more complex approaches, such as those based on the graphite oxide route. Furthermore, the present graphene materials exhibited good capacitive performance. These results arise from: (i) good wettability (relatively low solution resistance as shown in Figure 6 inset) and (ii) good exfoliation and adequate morphology (corrugated layers, with ample voids in-between as shown in Figure 4). The straightforward process to obtain the graphene products is due to the anodic exfoliation of graphite foil, which is a fast (40 min) and circular functionalization procedure, in which the phosphorus sources can be reused.

**Figure 7:**
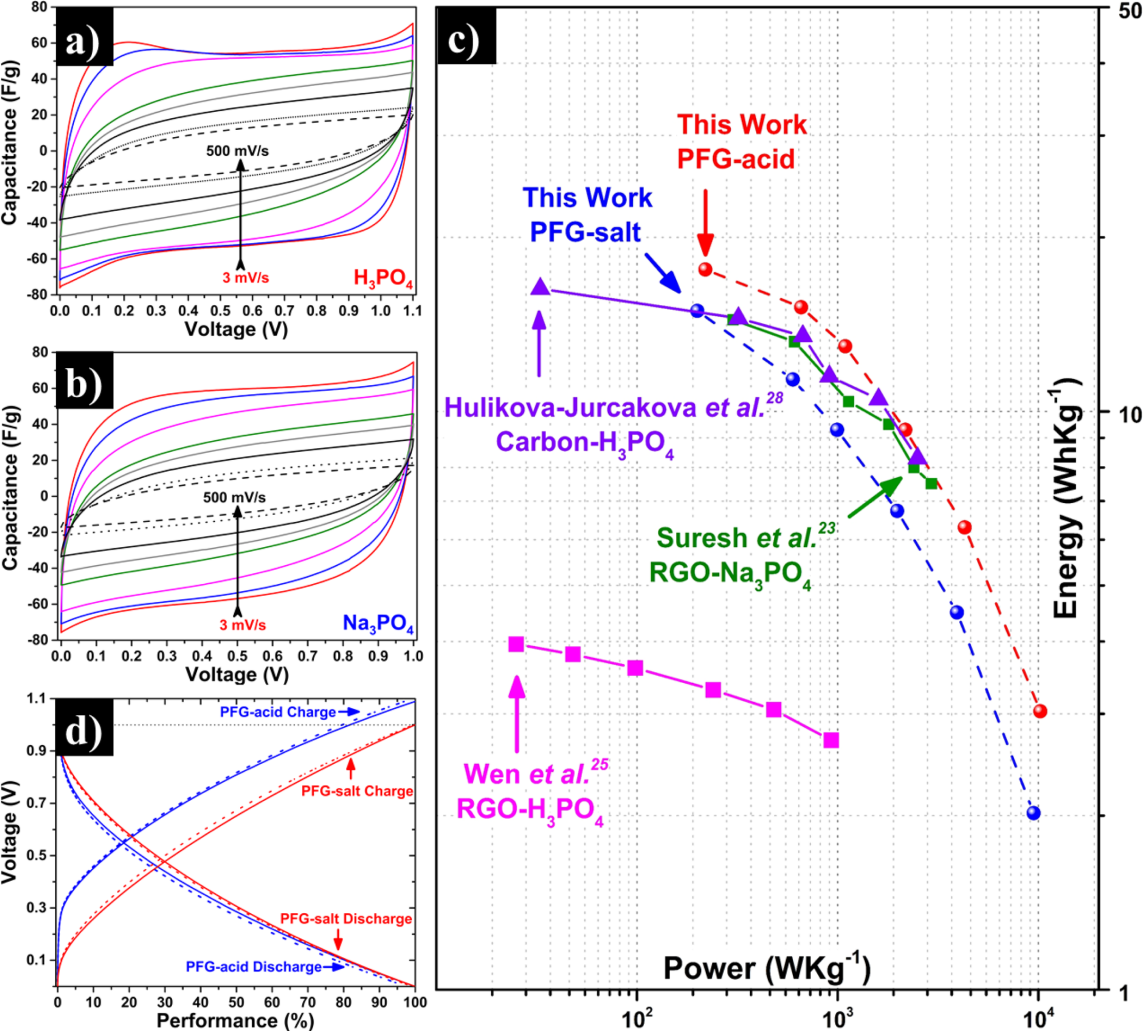
Energy-storage performance test for PFG-acid and PFG-salt (H_3_PO_4_ 0.25 M and Na_3_PO_4_ 0.05 M): VC curves (a) PFG-acid, (b) PFG-salt. (c) Ragone plot with representative carbon-derived structures phosphorus-doped and (d) cycling stability by galvanostatic charge–discharge at 2 A·g^–1^ at 1 cycle (line) and over 10,000 cycles (dot-line).

Since PFG-acid at 0.25 M and PFG-salt at 0.05 M were the investigated graphene materials with the higher specific capacitance, they were selected to assemble two-electrode symmetric devices. As shown in Figure 7a,b, the CV curves of the devices have a quasi-rectangular shape, demonstrating suitable electric double-layer capacitance performance. The good performance of the PFG electrodes was attributed to an appropriate wettability afforded by their chemical groups (phosphate and oxygen groups) and suitable morphology of corrugated nanosheets. At the same time, oxidation of the nanosheets is kept to moderate levels, thus avoiding excessive degradation of their electrical properties.

The performance of the PFG devices was investigated by GCD tests at different current densities at a voltage window of 1.1 and 1 V for PFG-acid and PFG-salt, respectively. In this case, the devices obtained using PFG-acid at 0.25 M possess energy and power density values up to 17.6 Wh·kg^–1^ (25.3 Wh·L^–1^, Figure S7) and 10,200 W·kg^–1^. On the other hand, the electrode with PFG-salt at 0.05 M yielded values of 14.9 Wh·kg^–1^ (21.3 Wh·L^–1^, Figure S7) and 9400 W·kg^–1^.

The gravimetric and volumetric energy and power densities were calculated using the total electrode mass and the electrode density (1.40–1.45 g·cm^–3^) together with the eqs S2 and S3. The results found here for the symmetric device compared favorably to results reported in the literature using carbon or graphene materials. For example, the power and energy densities delivered by the PFG materials were comparable to or even higher than those of phosphorus-doped graphene,^25^ as shown in Figure 7c. Other studies with functionalized graphene,^31,32^ or reduced graphene oxide^23^ have not reported results for two-electrode devices (only for their three-electrode counterparts).

The cycle life of an electrochemical device is a fundamental property for its practical application. As shown in Figure 7d the PFG-acid and PFG-salt preserve their performance over 10,000 cycles at 2 A·g^–1^ upon galvanostatic charge–discharge cycling. Specifically, the PFG-acid and PFG-salt products achieve a 98 and 99% of capacitance retention, respectively.

## Conclusions

We have successfully developed a straightforward approach to obtain phosphate-functionalized graphene (PFG) in a single-step via anodic exfoliation of graphite. Aqueous H_3_PO_4_ and Na_3_PO_4_ solutions were used as efficient phosphate sources and electrolytes. Suitable electrolyte concentrations that favor both exfoliations and the grafting of phosphate groups were identified and rationalized with phosphorous contents up to 2.2 at. % being obtained. The PFG-salt demonstrated a fast formation of the diffusion layer process, which means that these samples possess a better aqueous ion diffusion property at high density currents, than PFG-acid. The PFG materials were tested as an electrode material for capacitive energy storage in a two-electrode cell, achieving competitive energy values of around 17.6 Wh·kg^–1^ (25.3 Wh·L^–1^) and 14.9 Wh·kg^–1^ (21.3 Wh·L^–1^). Overall, the straightforward synthesis conditions as well as fast and facile recovery of electrolytes suggest a circular synthesis, promoting sustainable capacitive energy-storage devices, combined with a competitive performance in two-electrode symmetric devices and affordable synthesis.

## Supporting Information Available

Gravimetric capacitance; gravimetric energy density; gravimetric power density; percent composition and atomic ratios of phosphate-functionalized graphene (PFG) obtained with acid (H_3_PO_4_) or salt (Na_3_PO_4_) phosphate source at different concentrations by HR-XPS deconvolution; UV–vis spectroscopy corresponding to (a) PFG-acid (H_3_PO_4_) and (b) PFG-salt (Na_3_PO_4_) at different concentrations; the Nitrogen adsorption curves of the PFG-acid and PFG salt; scanning electron microscopy (SEM) and energy-dispersive X-ray spectroscopy (EDS) corresponding to (a–d) PFG-acid and (e–h) PFG-salt; cyclic voltammetry curves corresponding to PFG (a–c) acid (H_3_PO_4_) and (d–f) salt (Na_3_PO_4_) source at different concentrations and sweep rates; electrochemical impedance spectroscopy Bode Phase plots corresponding to (a) PFG-acid and (b) PFG-salt at different concentrations; galvanostatic charge–discharge profiles corresponding to PFG exfoliated with (a–c) acid (H_3_PO_4_) and (d–f) salt (Na_3_PO_4_) phosphate source at different concentrations and sweep rates; Ragone plot corresponding to representative-derived structures phosphorus doped (PDF)
