# Supplementary material for: A Simple and Expeditious Route to Phosphate-Functionalized, Water-Processable Graphene for Capacitive Energy Storage
Source: ACS Appl Mater Interfaces. 2021 Nov 9;13(46):54860–73. doi: 10.1021/acsami.1c12135 (PMC8631702; doi:10.1021/acsami.1c12135)
Supplement: Supplementary file 1 — am1c12135_si_001.pdf [file am1c12135_si_001.pdf]

Supporting Information

A simple and expeditious route to phosphate-functionalized, water-processable graphene for capacitive energy storage

Edgar H. Ramirez-Soria,[§] Sergio García-Dalí,[¥] Jose M. Munuera,[¥] Daniel F. Carrasco[¥] Silvia Villar-Rodil,[¥] Juan M. D. Tascón,[¥] Juan I. Paredes[¥,] and José Bonilla-Cruz[§,*]*

[§]Advanced Functional Materials & Nanotechnology Group. Centro de Investigación en Materiales Avanzados S. C. (CIMAV-Unidad Monterrey), Av. Alianza Norte 202, Autopista Monterrey-Aeropuerto Km 10, PIIT, C.P. 66628, Apodaca-Nuevo León, México. E-mail: jose.bonilla@cimav.edu.mx

[¥] Instituto de Ciencia y Tecnología del Carbono, INCAR-CSIC, C/Francisco Pintado Fe 26, 33011 Oviedo, Spain. E-mail: paredes@incar.csic.es

KEYWORDS: Phosphate-functionalized Graphene, Anodic Exfoliation, Capacitive Energy Storage.

The gravimetric capacitance (C_s, F g^{-1}) of a single electrode (three-electrode) and both[1,2]

$$C_s = \frac{2\ I\ t_D}{m\ \Delta V_D} \qquad \textbf{Equation S1}$$

Where I (A) is the current, t_D (s) is the time of discharge without internal resistance, m (g) the active material mass and ΔV_D is the potential difference (V) over-discharge process without internal resistance. The mass used for two-electrode configuration was the sum of the active material mass from two electrodes. The gravimetric energy density (E, Wh Kg^{-1}) was calculated trough **Equation S2**:[2]

$$E = \frac{C_S\ \Delta V_D^2}{2*3.6} \qquad \textbf{Equation S2}$$

Where Cs is the gravimetric capacitance (F g^{-1}, previously calculated) and ΔV_D is the potential difference (V) for the discharge process without internal resistance. Finally, the gravimetric power density (P, W Kg^{-1}) was calculated by **Equation S3**:[1,3]

$$P = 3600 * \frac{E}{\Delta t_D} \qquad \textbf{Equation S3}$$

Table S1. Percent composition and atomic ratios of phosphate-functionalized graphene (PFG) obtained with acid (H_3PO_4) or salt (Na_3PO_4) phosphate source at different concentrations by HR-XPS deconvolution.

	**[H_3PO_4]**			**[Na_3PO_4]**		
	**0.25M**	**0.50M**	**1M**	**0.05M**	**0.10M**	**0.25M**
**C-sp^2**	40.7	48.1	65.4	46.1	46.9	53.9
**C-sp^3**	25.1	22.2	10.5	21.8	22.3	17.1
**C-O**	17.0	15.7	16.2	16.2	16.0	17.1
**C-O-C**	8.0	7.0	3.0	8.3	8.0	5.0
**C=O**	2.0	1.5	1.6	2.1	1.9	1.5
**O-C=O**	7.2	5.5	3.3	5.5	4.9	5.4
**C1s**	76.3	81.6	86.7	80.7	82.8	86.9
**O1s**	21.5	17.0	12.2	17.9	16.1	12.7
**P2p**	2.2	1.4	1.1	1.4	1.1	0.4
**O/C (C1s)**	0.37	0.32	0.26	0.33	0.32	0.32
**O/C[a]**	0.28	0.21	0.14	0.22	0.19	0.15
**O/C[b]**	0.17	0.14	0.09	0.15	0.14	0.13
**P/C**	0.029	0.017	0.013	0.017	0.013	0.005

[a]With phosphate functional groups

[b]Without phosphate functional groups

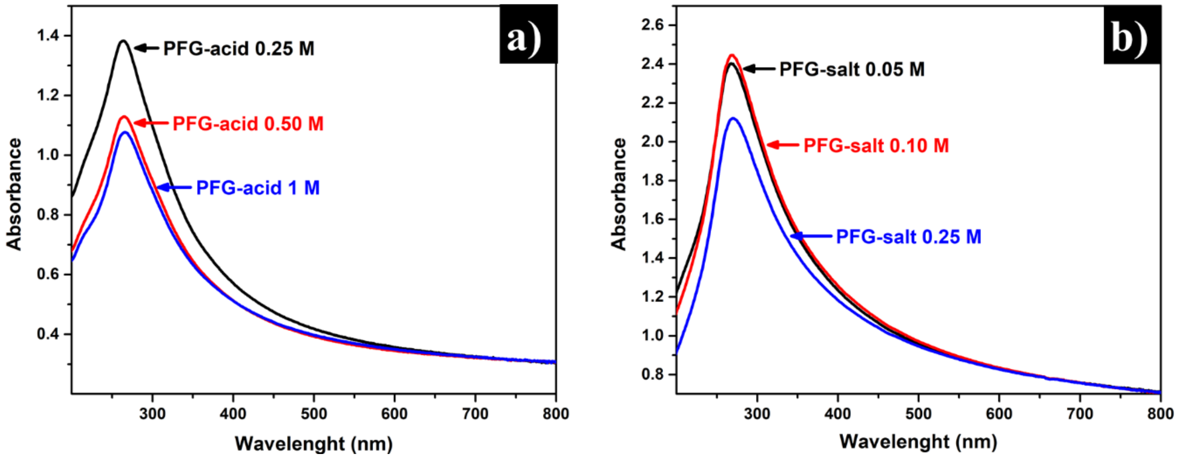

Figure S1. UV-vis spectroscopy corresponding to a) PFG-acid (H_3PO_4) and b) PFG-salt (Na_3PO_4) at different concentrations.

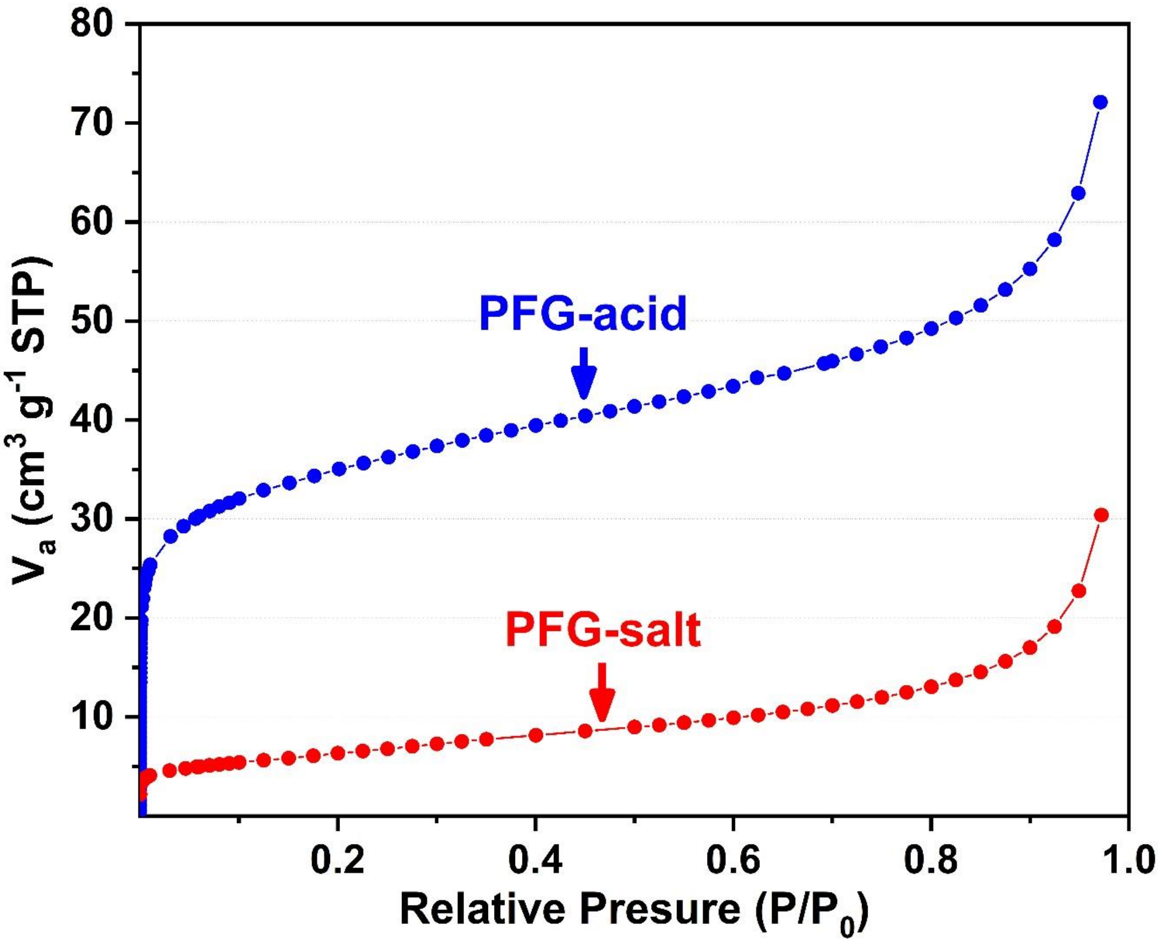

Figure S2. The Nitrogen adsorption curves of the PFG-acid and PFG salt

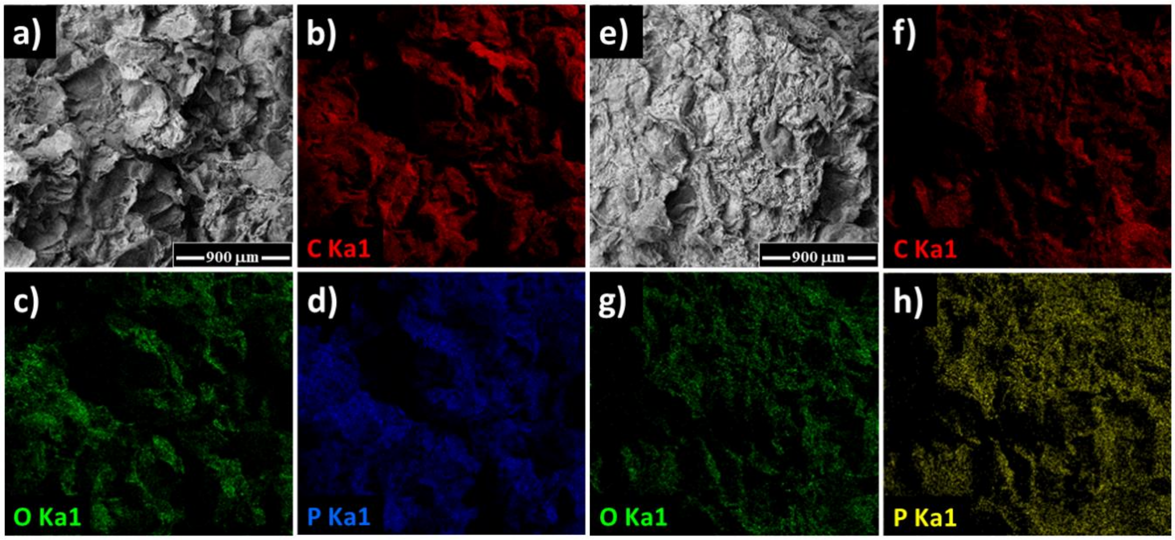

Figure S3. Scanning electron microscopy (SEM) and energy-dispersive X-ray spectroscopy (EDS) corresponding to (**a-d**) PFG-acid and (**e-h**) PFG-salt.

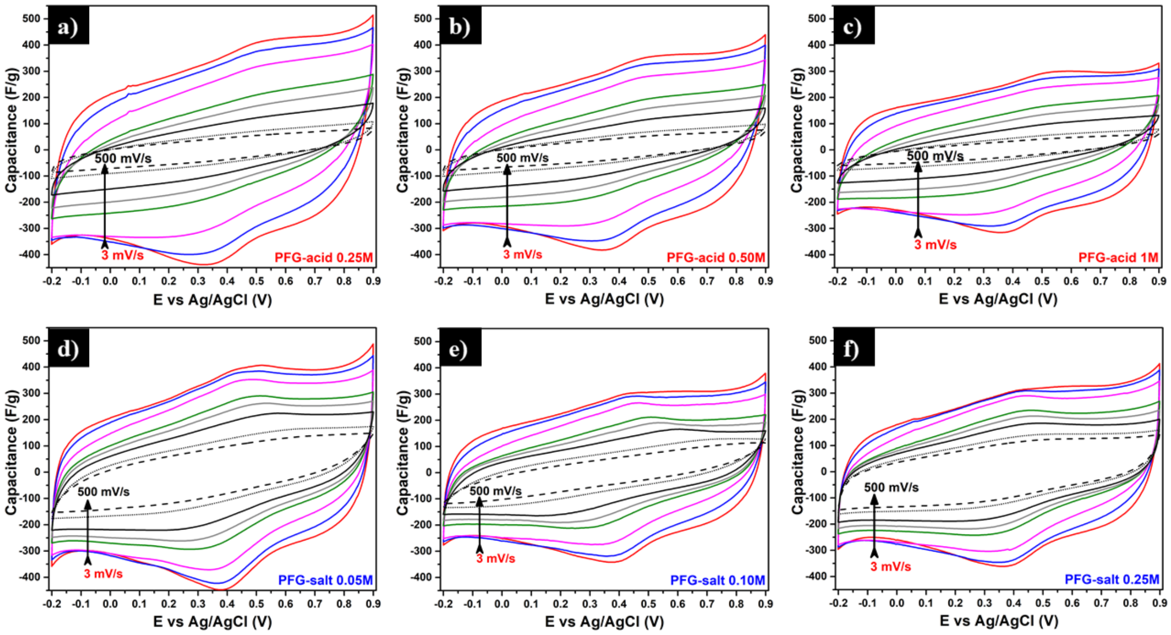

Figure S4. Cyclic voltammetry curves corresponding to PFG (**a-c)** acid (H_3PO_4) and **(d-f)** salt (Na_3PO_4) source at different concentrations and sweep rates.

Figure S4 shows the cyclic voltammetry corresponding to PFG-acid and PFG-salt at different concentrations and sweep rates. The CV curves exhibit an almost rectangular shape for voltage windows of 1.1 V (-0.2 – 0.9 V vs Ag/AgCl) and the pseudo-capacitive reactions around 0.4 V (vs Ag/AgCl). In both PFG-acid and PFG-salt samples the specific capacitance (voltammetric current) decreased over phosphorous content was decreased too.

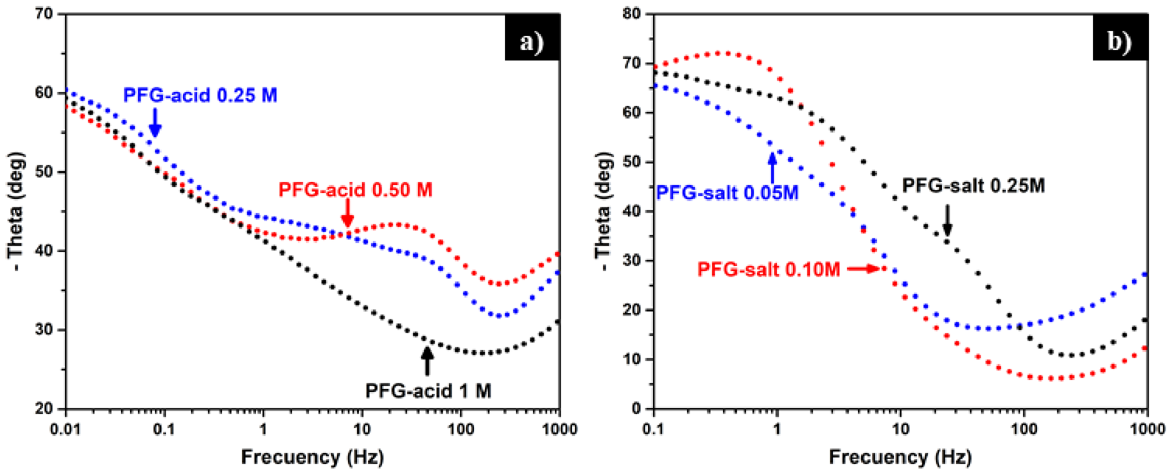

Figure S5. Electrochemical impedance spectroscopy Bode Phase plots corresponding to **a)** PFG-acid and **b)** PFG-salt at different concentrations.

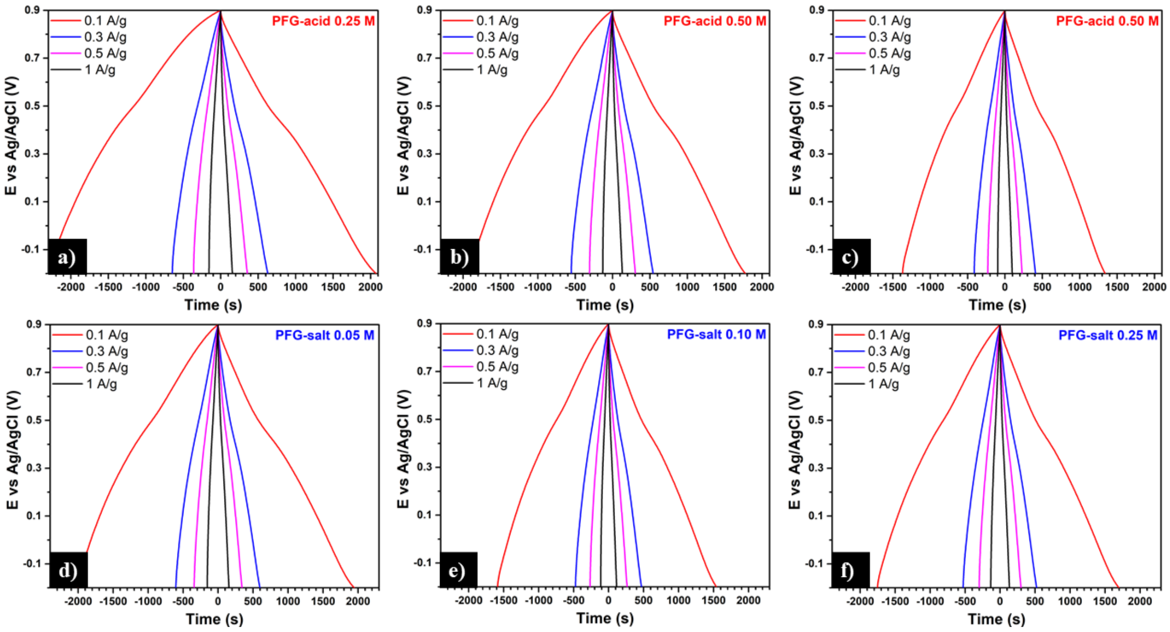

Figure S6. Galvanostatic charge-discharge profiles corresponding to PFG exfoliated with **(a-c)** acid (H_3PO_4) and **(d-f)** salt (Na_3PO_4) phosphate source at different concentrations and sweep rates.

Figure S6 shows the GC profiles which are in good concordance with CV curves, the electrochemical activity is dependent on phosphorous content and oxidation level.

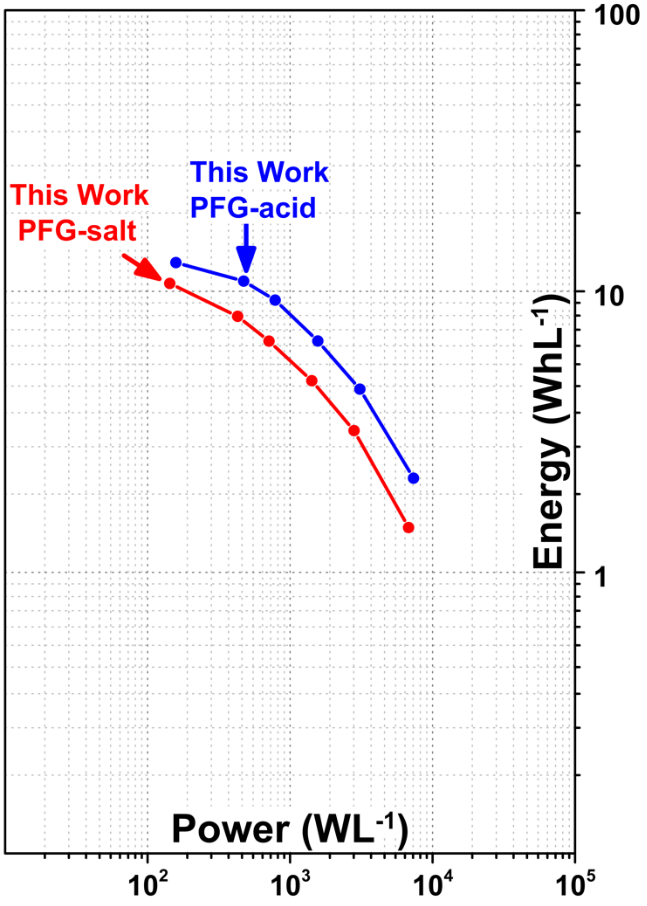

Figure S7. Ragone plot corresponding to representative-derived structures phosphorus doped.

REFERENCES.

(1) Bi, Z.; Huo, L.; Kong, Q.; Li, F.; Chen, J.; Ahmad, A.; Wei, X.; Xie, L.; Chen, C. Structural Evolution of Phosphorus Species on Graphene with a Stabilized Electrochemical Interface. *ACS Appl. Mater. Interfaces* **2019**, *11* (12), 11421–11430. https://doi.org/10.1021/acsami.8b21903.

(2) Chee, W. K.; Lim, H. N.; Zainal, Z.; Huang, N. M.; Harrison, I.; Andou, Y. Flexible Graphene-Based Supercapacitors: A Review. *J. Phys. Chem. C* **2016**, *120* (8), 4153–4172. https://doi.org/10.1021/acs.jpcc.5b10187.

(3) Munuera, J. M.; Paredes, J. I.; Enterría, M.; Pagán, A.; Villar-Rodil, S.; Pereira, M. F. R.; Martins, J. I.; Figueiredo, J. L.; Cenis, J. L.; Martínez-Alonso, A.; et al. Electrochemical Exfoliation of Graphite in Aqueous Sodium Halide Electrolytes toward Low Oxygen Content Graphene for Energy and Environmental Applications. *ACS Appl. Mater. Interfaces* **2017**, *9* (28), 24085–24099. https://doi.org/10.1021/acsami.7b04802.
